# Knowledge and Awareness of Enhanced Recovery After Surgery (ERAS) Protocols Among Anesthesiologists: A Multicenter Cross-Sectional Survey

**DOI:** 10.3390/healthcare14172690

**Published:** 2026-08-24

**Authors:** Beste Mutlu Daglioglu, Damla Kaytanci Ozcelik, Aysenur Sumer Coskun, Oyku Irem Subasi

**Affiliations:** Department of Anesthesiology and Reanimation, Gulhane Faculty of Medicine, University of Health Sciences, Antalya City Hospital, Antalya 07080, Turkey

**Keywords:** anesthesiology, enhanced recovery after surgery, knowledge assessment, perioperative medicine, physician survey, postoperative care

## Abstract

Background: Enhanced Recovery After Surgery (ERAS) protocols aim to reduce surgical stress and optimize postoperative recovery, yet anesthesiologists’ knowledge of these protocols remains incompletely characterized. Methods: This cross-sectional survey (May–August 2025) evaluated knowledge of general and surgery-specific ERAS protocols among anesthesiology specialists, faculty, and residents in Turkey using a 37-item questionnaire based on current ERAS Society guidelines; the primary outcome was the continuous knowledge score, with Bloom’s cutoffs applied descriptively. Results: Among 380 participants (72.4% specialists/faculty, 27.6% residents), the mean standardized score was 68.5 ± 10.3; 47.6% (95% CI 42.7–52.7%) and 28.2% (95% CI 23.9–32.9%) had moderate and high knowledge, respectively. Professional title was independently associated with knowledge: scores were higher among specialists and faculty than residents (β = 5.92 and 6.79), and residents were less likely than specialists (OR 0.423) or faculty (OR 0.344) to fall in a higher knowledge category. Preoperative knowledge was lower than intraoperative/postoperative scores. Module-specific scores, interpreted descriptively because modules differ in item difficulty and subspecialty exposure, were highest in bariatric (74.6%) and lowest in colorectal surgery (49.8%). Conclusions: Anesthesiologists demonstrated predominantly moderate theoretical ERAS knowledge, with gaps in preoperative optimization and specific modules, indicating that curricula and continuing education may benefit from greater emphasis on these areas.

## 1. Introduction

Enhanced Recovery After Surgery (ERAS) protocols are evidence-based, multidisciplinary perioperative care models designed to mitigate the physiological stress response to surgery and accelerate postoperative recovery. ERAS principles are standardized through current guidelines specific to various surgical fields and encompass practices such as preoperative optimization, multimodal and opioid-sparing analgesia, goal-directed fluid management, maintenance of normothermia, and early mobilization [1,2].

High adherence to ERAS guidelines improves outcomes and reduces healthcare costs [3,4,5,6], yet real-world implementation remains inconsistent across centers—a heterogeneity frequently attributed to variable levels of knowledge and awareness among perioperative staff internationally [7,8,9,10]. Anesthesiologists play a critical role across all ERAS-covered surgical disciplines [3,11], but comprehensive data describing the extent to which anesthesiologists master current, guideline-specific ERAS recommendations remain limited and inconsistent even across well-resourced healthcare systems, particularly outside North American and Western European settings [8,12,13].

This study aimed to evaluate the knowledge and awareness of anesthesiologists practicing across Turkey regarding both general and surgery-specific ERAS protocols, and to objectively identify subtopics where knowledge gaps exist. By anchoring survey content directly in current ERAS Society guideline recommendations, the study further sought to prompt participating physicians to reflect on their routine clinical practice, and to provide an evidence base for designing targeted educational interventions within this and comparable healthcare settings.

With respect to the secondary endpoint comparing knowledge levels across professional and demographic characteristics, the following a priori hypothesis was specified: H1—ERAS knowledge level differs significantly according to professional title (resident, specialist, faculty member); H0—ERAS knowledge level does not differ according to professional title.

## 2. Materials and Methods

### 2.1. Study Design and Ethical Approval

This study was designed as an observational-descriptive, multicenter cross-sectional survey using a convenience/snowball sample and is reported in accordance with the STROBE statement for cross-sectional studies (Table S1). Approval was obtained from the Clinical Research Ethics Committee of the University of Health Sciences, Antalya City Hospital (Date: 8 May 2025, Decision No: 6/38). The study complied with the Declaration of Helsinki, and electronic informed consent was obtained from all participants. No personally identifying information (e.g., name or national identification number) was collected; responses were recorded anonymously and stored on a password-protected institutional server accessible only to the research team.

### 2.2. Population and Sample

The study population consisted of anesthesiology residents, specialists, and faculty members practicing across university, training-research, state, and private hospitals in Turkey. Based on Turkish Society of Anesthesiology and Reanimation (TARD) registry data (4274 specialists/faculty and 5502 residents), the target population was approximately 9776 physicians; as TARD membership is not mandatory, this figure may not capture all licensed anesthesiologists nationally, and the true denominator could differ somewhat from this estimate. The minimum required sample size was calculated a priori using G*Power version 3.1 (Heinrich Heine University Düsseldorf, Düsseldorf, Germany). Assuming a Type I error (α) of 0.05, a statistical power of 0.80, and a conservatively chosen small-to-medium effect size (Cramer’s V = 0.20, in the absence of comparable prior effect-size data from Turkish anesthesiologists), the minimum required total sample size was 352 participants, sufficient to power the planned subgroup comparisons of knowledge level across professional title, experience, and institution type; the study was designed to exceed this threshold. As a cross-check using Cochran’s formula for a known population (N = 9776; 95% confidence level; 5% margin of error; *p* = 0.5), the minimum sample size was independently estimated at approximately 370, consistent with the G*Power-based calculation above and with the sample-size approach used in a comparable prior ERAS knowledge survey among Turkish anesthesiology trainees [8].

**Figure 1:**
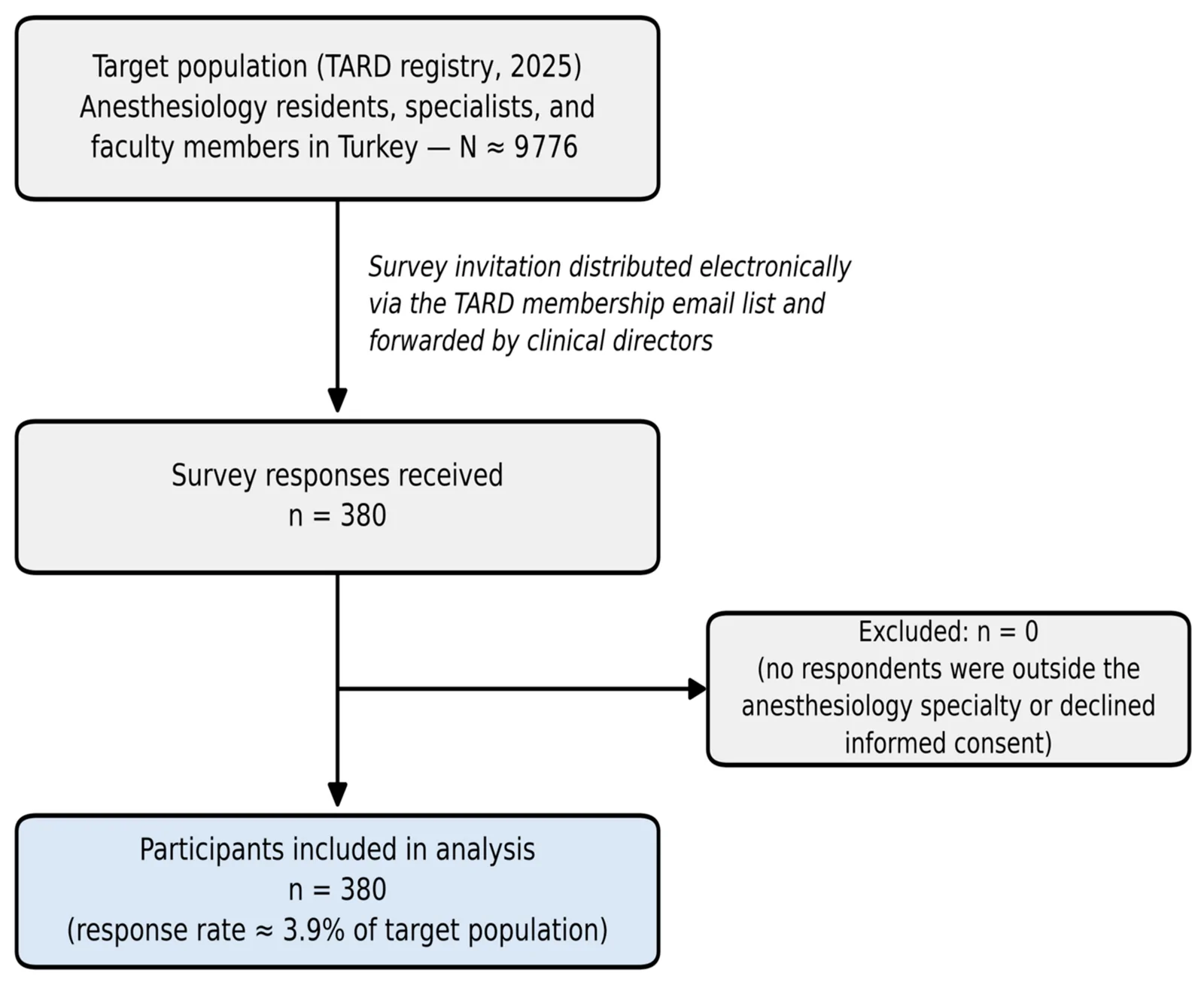
Participant flow diagram. The digital distribution format precluded verification of the exact number of individuals who received or opened the survey invitation (e.g., due to outdated email addresses or spam filtering); the response rate shown should therefore be interpreted as a conservative estimate rather than a precise denominator.

A combination of purposive-convenience and snowball sampling was used: invitations were distributed electronically to the full TARD membership list and forwarded by clinical directors, with participants encouraged to share the link further; consequently, the 9776 figure should be regarded as an approximate rather than an exact denominator. Physicians outside the anesthesiology specialty or without informed consent were to be excluded. A total of 380 responses were received, all meeting eligibility criteria, with no exclusions (Figure 1). This corresponds to a response rate of approximately 3.9% relative to the target population, which should not be interpreted as indicating representativeness given the sampling method. This non-probability approach was adopted in the absence of a centralized, complete contact registry for anesthesiologists in Turkey, and is inherently susceptible to selection bias, as discussed further in the Limitations.

### 2.3. Data Collection Tool

A structured questionnaire was developed by the researchers based on current 2023–2025 ERAS Society guidelines. Content and clarity were reviewed by five ERAS-experienced anesthesiology specialists (minimum five years’ experience; two faculty members), leading to minor refinements in item wording without altering content domains. A pilot study in 15 anesthesiology and reanimation physicians led to removal of two confusing, overlapping response options: a 3 h option for breast milk fasting duration (given divergent European/American guideline recommendations) and an 8 h option for solid/fatty food fasting duration. The complete 37-item questionnaire, translated into English with the correct answer for each item indicated, is provided in File S1.

The final survey included an informed consent section, sociodemographic items (age, sex, professional title, experience, institution type), and 37 knowledge-assessment questions covering general perioperative principles (preoperative, intraoperative, and postoperative) and ten surgical modules (bariatric, 2 items; cardiovascular [comprising cardiac and vascular surgery items], 5; colorectal, 3; hepatic, 2; spinal, 1; neonatal, 1; orthopedic, 3; thoracic, 1; radical cystectomy, 1; and emergency laparotomy, 4), reflecting the breadth of ERAS-relevant surgical practice encountered in a typical tertiary anesthesiology department. Independently of this module structure, each of the 37 items was assigned a priori to the perioperative phase to which its content applies, so that the two classifications are overlapping rather than mutually exclusive and the phase and module analyses were performed separately: preoperative (items 1–4, 11–13, 17–19, 21, 22, and 37; 13 items), intraoperative (items 14–16, 24, 25, 27, 29, 30, and 34; 9 items), and postoperative (items 5–10, 20, 23, 26, 28, 31–33, 35, and 36; 15 items).

Correct answers were coded as 1 point and incorrect as 0 points; as all items were mandatory fields in the online platform (Google Forms), no responses were missing. Raw scores were standardized to a 0–100 scale and categorized using Bloom’s cutoff points, a scheme widely used in knowledge/KAP survey methodology [14]: <60 “low”, 60–74 “moderate”, and ≥75 “high” knowledge, the upper threshold consistent with a modified adaptation reported elsewhere [15]. These a priori cutoffs have not been independently validated for ERAS knowledge assessment (see Limitations).

### 2.4. Data Collection Process

Data were collected between 10 May and 1 August 2025, exclusively via an online platform (Google Forms). The survey link was distributed through the TARD membership list, professional communication groups, and clinical directors at participating institutions across multiple regions of Turkey, in order to capture a heterogeneous sample spanning diverse geographical areas and institution types; participation was restricted to one response per session to prevent duplicate entries.

### 2.5. Endpoints

The primary outcome measure was the continuous standardized ERAS knowledge score (0–100); Bloom’s categories were applied as a descriptive convention and served as the outcome variable of the ordinal regression model. Secondary endpoints included comparing knowledge levels across perioperative phases (preoperative, intraoperative, postoperative), surgical sub-disciplines, and participants’ professional and institutional characteristics. A full description of the dependent and independent variables, their roles in the analyses, and their measurement scales is provided in Table S2.

### 2.6. Statistical Analysis

Statistical analyses were performed using Jamovi (v2.7; The Jamovi project, Sydney, Australia); internal consistency was assessed with the Kuder–Richardson 20 (KR-20) coefficient, though the heterogeneous, ten-module item set was not intended as a unidimensional scale.

Normality was assessed via Shapiro–Wilk testing, histograms, and Q-Q plots; as variables were non-normally distributed, descriptive statistics were reported as medians (IQR), and categorical variables as frequencies and percentages. The distribution of the total knowledge score was nonetheless approximately symmetric (mean 68.5, median 67.6), and both the mean ± SD and the median (IQR) are therefore reported for this variable.

Two-group comparisons used the Mann–Whitney U test (rank biserial effect size); comparisons among ≥3 groups used Kruskal–Wallis with Bonferroni-corrected Dunn post hoc tests. The Friedman test compared dependent measurements (perioperative phases, surgical modules) with Durbin-Conover post hoc analysis; modules represented by a single item (spinal, neonatal, thoracic, radical cystectomy) were excluded from module-level comparison, leaving six modules with sufficient item coverage (see Section 2.3 for the item count of each module). Categorical associations were assessed via Pearson’s Chi-Square test (Cramer’s V), and Ordinal Logistic Regression (professional title and institution type as covariates) identified independent correlates of knowledge category (the proportional odds assumption was verified); a parallel linear regression modeled the continuous knowledge score, and the two models are reported as complementary rather than hierarchical. Sample representativeness (professional-title distribution) was assessed against TARD registry proportions via a chi-square goodness-of-fit test. Significance was set at *p* < 0.05.

## 3. Results

The overall KR-20 reliability coefficient was 0.651. Of 380 participants, most were specialists (54.7%), followed by residents (27.6%) and faculty members (17.6%), predominantly working in training-research (37.6%) and university hospitals (30.8%). Demographic and professional characteristics are presented in Table 1.

Compared with national TARD registry proportions (56.3% residents vs. 43.7% specialists/faculty), the sample’s professional-title distribution diverged significantly (χ^2^(1) = 126.8; *p* < 0.001; w = 0.58), with specialists and faculty members markedly overrepresented (72.4% of the sample vs. 43.7% nationally); this divergence is examined further in the Limitations.

The continuous knowledge score (mean 68.5 ± 10.3, median 67.6, IQR 62.2–75.7) constituted the primary outcome measure. Using Bloom’s classification as a descriptive convention, 47.6% (95% CI 42.7–52.7%) of participants demonstrated moderate, 28.2% (95% CI 23.9–32.9%) high, and 24.2% (95% CI 20.2–28.8%) low knowledge, with the sample clustering primarily at the moderate level.

Professional title was significantly associated with knowledge level (χ^2^(4) = 22.6; *p* < 0.001; Cramer’s V = 0.173), with residents showing a markedly higher proportion of low knowledge and a lower proportion of high knowledge than specialists or faculty members (Table 2).

Subgroup analyses showed residents with 2–5 years’ experience scored higher than those with 0–2 years (Mann–Whitney U = 660; *p* < 0.001; rank biserial = 0.508), while specialists with 0–10 years scored higher than those with >10 years (an associative finding) (U = 4232; *p* = 0.040; rank biserial = −0.169) (Table 2).

Institution type was also significantly associated with knowledge level (χ^2^(6) = 13.3; *p* = 0.038; Cramer’s V = 0.132), with state hospital physicians showing the highest proportion of high knowledge (42.3%), while training-research and university hospital physicians predominantly fell into the moderate category (Table 2).

Ordinal logistic regression identified professional title as an independent correlate of knowledge level: residents were significantly less likely than specialists (OR = 0.423; 95% CI 0.242–0.736; *p* = 0.002) or faculty members (OR = 0.344; 95% CI 0.189–0.620; *p* < 0.001) to be in a higher knowledge category; institution type showed no independent effect (all *p* > 0.05). The continuous score (mean 68.5 ± 10.3, median 67.6, IQR 62.2–75.7) yielded concordant results in a parallel linear regression model: title remained independently associated with score (specialists: β = 5.92, *p* < 0.001; faculty: β = 6.79, *p* < 0.001), while institution type remained non-significant (all *p* > 0.25; R^2^ = 0.086, *p* < 0.001), supporting the robustness of the categorical analysis. The model nonetheless explained only a modest proportion of the variance in knowledge score, indicating that most variation is not accounted for by the characteristics measured here.

**Figure 2:**
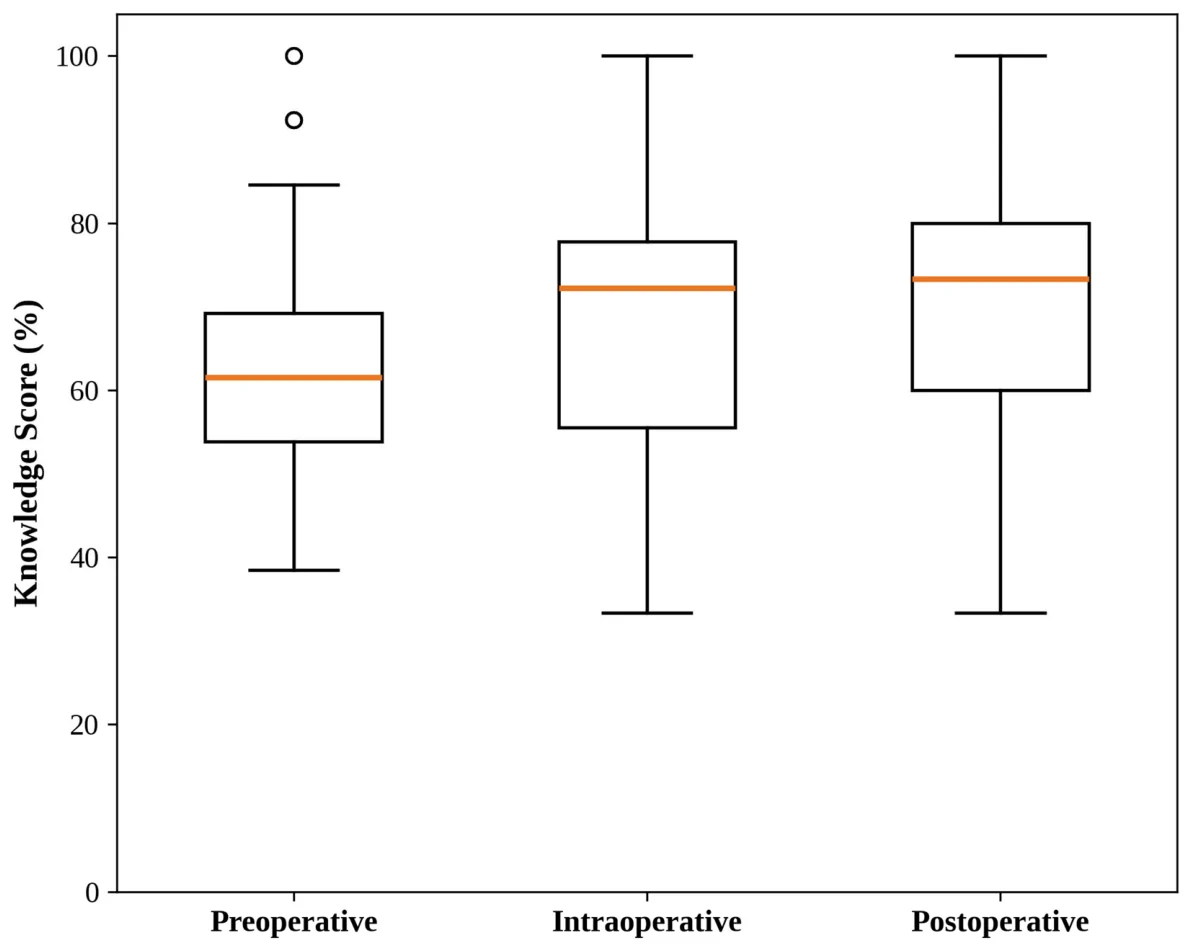
Comparison of ERAS knowledge percentages according to preoperative, intraoperative, and postoperative phases.

Knowledge differed significantly across perioperative phases (Friedman χ^2^(2) = 56.9; *p* < 0.001): preoperative scores were lower than intraoperative and postoperative scores (both *p* < 0.001), which did not differ from each other (*p* = 0.079) (Figure 2).

**Figure 3:**
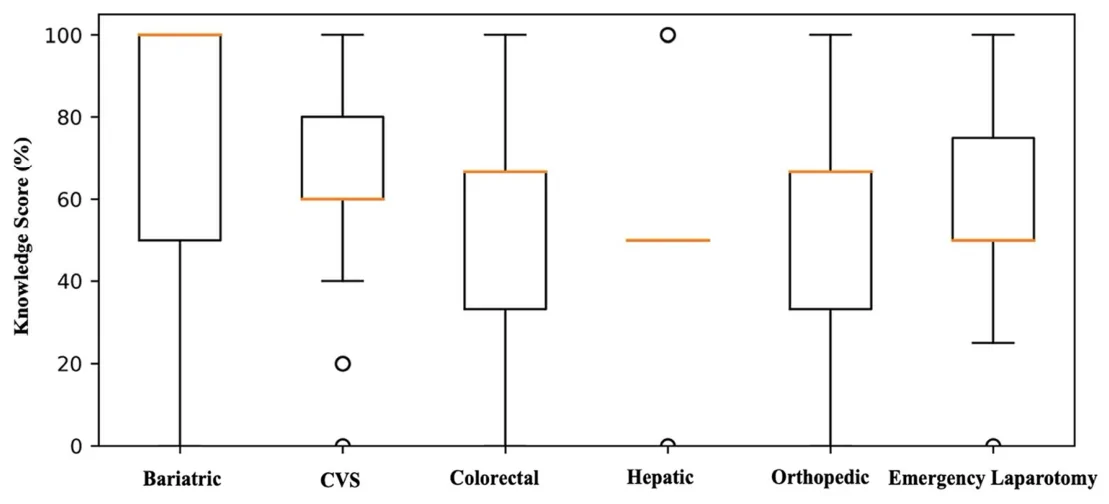
ERAS knowledge scores (%) across surgical specialties. Spinal, neonatal, thoracic, and radical cystectomy modules, each represented by a single knowledge item, were excluded from this module-level comparison. CVS: cardiovascular surgery.

Among the six surgical modules with sufficient item coverage for comparison, module-specific standardized scores differed significantly (Friedman χ^2^(5) = 170.0; *p* < 0.001); scores were highest in bariatric surgery (74.6%), followed by cardiovascular (67.6%) and orthopedic surgery (61.8%), and lowest in emergency laparotomy (60.1%), hepatic surgery (58.9%), and colorectal surgery (49.8%) (Figure 3). As these modules differ in item count, item difficulty, and likely subspecialty exposure, these differences are reported descriptively rather than as a ranked comparison of module-specific knowledge.

Item-level distributions are summarized in Table 3. Four items showed markedly low correct response rates (<30%)—timing of pregabalin administration before cardiac surgery, goal-directed fluid therapy in lumbar fusion, target preoperative HbA1c for cardiac surgery, and oral carbohydrate loading in diabetic patients—predominantly specialty-specific pharmacological or hemodynamic items rather than general principles. The highest correct response rates were recorded for hypoalbuminemia risk in cardiac surgery, solid-food fasting duration, and orthopedic multimodal analgesia. Corresponding percentages are given in Table 3. Within general principles, knowledge was heterogeneous: breast milk fasting duration (83.4%) scored lower than clear-fluid fasting (92.1%), and fewer than two-thirds of participants correctly identified that smoking, advanced age, and obesity are not among the classic Apfel PONV risk factors (58.9–64.7% correct).

## 4. Discussion

This survey provides a detailed assessment, in a multicenter convenience sample, of anesthesiologists’ theoretical ERAS knowledge in Turkey, examining its distribution across levels of seniority and clinical experience.

The KR-20 coefficient (0.651) falls below the conventional 0.70 threshold. This is unsurprising given the multidimensional, ten-module structure of the questionnaire, but it should not be read as evidence of adequate reliability: it reflects internal consistency only and does not substitute for construct validity or test–retest reliability, neither of which was formally assessed prior to the survey’s distribution; this psychometric limitation should be borne in mind when interpreting all findings reported below (see Limitations).

Overall scores clustered at a moderate level, consistent with prior literature: Jiang et al. reported ~65% average ERAS knowledge among anesthesiologists [16], and Haseeb et al. found only partial guideline familiarity in 45.3% of perioperative clinicians [7]. This suggests baseline familiarity with ERAS concepts without full integration of guideline-specific detail. As adherence to ERAS guidelines in major surgery reduces hospitalization, cost, and complications [4,5,6], the knowledge gap identified here may represent a potential barrier to realizing these benefits in practice, though clinical adherence itself was not assessed.

Successful ERAS implementation depends on shared theoretical grounding across the multidisciplinary team of surgeons, anesthesiologists, and nurses. Existing literature highlights inconsistent knowledge levels among surgical specialists regarding current protocols [7,17,18,19,20] and perioperative nurses similarly exhibit theoretical deficits in ERAS awareness, underscoring the need for structured institutional training [21,22,23,24]. Assessing the knowledge base of anesthesiologists, who are integral to every phase of perioperative care, is therefore useful for identifying knowledge gaps that may inform future educational interventions, rather than for directly characterizing clinical practice deficiencies.

Professional title was independently associated with knowledge, with residents showing a markedly higher proportion of low-knowledge classification than specialists or faculty (Table 2), suggesting substantial gains during the transition from residency to specialization. Specialists with ≤10 years’ experience scored higher than more senior colleagues (an associative finding), possibly reflecting closer familiarity with contemporary, frequently updated guidelines. This mirrors prior findings linking greater clinical experience and higher title with superior ERAS knowledge [7,16].

Item-level analysis revealed strong performance on routine topics (solid-food fasting, 96.6%; multimodal analgesia, 96.1%) but poor performance on some specialty-specific items. While goal-directed fluid therapy is a critical, well-established component of gastrointestinal ERAS pathways [2,25,26], only 20.3% correctly recognized that its routine application is not specifically supported for limited-extent procedures such as 1–2 level lumbar fusion [27,28]; the high rate of incorrect (‘recommended’) responses may reflect an inclination toward cautious fluid monitoring in this context, rather than a true knowledge deficit. A similarly charitable reading may apply to the low correct-response rate for timing of pregabalin administration before cardiac surgery (13.7%), as recommended timing varies across enhanced-recovery protocols and institutional practices, and this item may therefore reflect the absence of a single, uncontested guideline standard rather than a true knowledge gap. In contrast, only 20.5% identified the correct preoperative HbA1c target for cardiac surgery, despite established evidence linking hyperglycemia to postoperative complications [29,30], consistent with previously reported gaps in cardiac-specific ERAS knowledge [12]. A comparable gap was apparent in preoperative nutritional preparation: only 27.4% of participants answered the item on oral carbohydrate loading in patients with diabetes in accordance with the guideline used as the reference standard for this item [30]. The colorectal module—the original focus of ERAS—recorded the lowest overall score (49.8%), with only 41.3% identifying thoracic epidural analgesia as the gold-standard technique [2], although, as noted above, between-module comparisons confound knowledge with item difficulty and subspecialty exposure. These findings are consistent with prior work showing strong general but weak module-specific knowledge [16].

Lower preoperative than intraoperative/postoperative knowledge may reflect a relatively greater clinical focus on intraoperative and immediate postoperative care among anesthesiologists, as preoperative optimization tasks are often shared with or delegated to surgical or nursing teams; this interpretation, however, was not directly tested in the present study. This pattern echoes prior reports of anesthesiologist strength in intraoperative/postoperative domains alongside preoperative nutritional-planning deficits [12], and findings that preoperative optimization is the most difficult ERAS phase to implement clinically [18].

This study has several strengths, including its comparatively large, multi-institutional sample; a survey instrument developed directly from current ERAS Society guidelines and refined through expert review and pilot testing; comprehensive coverage of both general perioperative principles and ten distinct surgical modules; and the use of complementary categorical and continuous analytic approaches, which yielded convergent findings and supported the robustness of the primary conclusions.

Several limitations warrant consideration. As a single-country study, our findings reflect the healthcare, training, and guideline-implementation context specific to Turkey, and generalization to other practice environments should be made cautiously; given that Turkey follows an internationally recognized (ESAIC) anesthesiology training curriculum, the identified knowledge patterns may nonetheless be informative for comparable training systems, pending confirmatory studies elsewhere. This study assessed theoretical knowledge rather than actual clinical adherence, which was not measured and may differ from the results reported here. Convenience/snowball sampling and self-selection—favoring physicians with a preexisting interest in ERAS—may have overestimated true population knowledge, and the a priori sample-size calculation addresses only the precision of the resulting estimates, not the representativeness of who responded. Because the survey link was forwarded and shared beyond the original distribution list, the number of individuals who received or opened it could not be determined; responders could therefore not be compared with non-responders, and non-response bias cannot be assessed—a limitation distinct from the sampling skew described next, and one that also makes the calculated response rate a conservative estimate. To test that skew formally, we compared the sample with national registry data: specialists and faculty members were significantly and markedly overrepresented relative to national TARD demographics (χ^2^(1) = 126.8; *p* < 0.001; w = 0.58), comprising nearly three-quarters of the sample versus 43.7% nationally; this divergence, together with the low response rate, meaningfully limits generalizability to the broader population of Turkish anesthesiologists, and residents in particular are likely underrepresented. The questionnaire’s psychometric properties (Content Validity Index, construct validity, test–retest reliability) were not formally established prior to its distribution to the target population, beyond expert content review and pilot testing—as no previously validated instrument existed for this purpose, and given the resource and time constraints associated with a survey of this scope, these steps were prioritized over full psychometric validation, consistent with common practice in the development of new knowledge/KAP survey instruments; Bloom’s cutoff thresholds were determined a priori and are not independently validated for ERAS assessment; and for items where international guidelines diverge (e.g., breast milk fasting duration), a single reference standard was used, potentially penalizing guideline-concordant alternative answers. The restricted number of questions per surgical module limited in-depth analysis within specific areas, consistent with the known instability of internal consistency estimates for scales with few items [31]. Finally, given the cross-sectional design, associations with professional or institutional characteristics should be interpreted as associative, not causal.

Taken together, these findings suggest that closing ERAS-related knowledge gaps among anesthesiologists will likely require more than generic awareness campaigns. Structured, module-specific educational content, embedded longitudinally within residency training and reinforced through continuing medical education, may be better suited to addressing the specialty-specific deficits identified here than a single, broad curriculum update; content prioritizing the preoperative phase, potentially delivered through dedicated preoperative optimization clinics, would be a reasonable starting point, and such continuing medical education (CME) provision may help standardize knowledge across training levels and institution types. Notably, the gaps identified here persisted despite Turkey’s adoption of the ESAIC curriculum. Future prospective research directly linking such educational interventions to measured changes in clinical adherence and patient outcomes would help establish whether improved theoretical knowledge translates into tangible perioperative benefits.

## 5. Conclusions

In this multicenter convenience sample, anesthesiologists demonstrated predominantly moderate theoretical ERAS knowledge, with notable gaps in preoperative optimization and, descriptively, in specific modules such as colorectal surgery. As these findings reflect knowledge and awareness rather than observed practice, they suggest residency curricula and continuing education may benefit from greater emphasis on these areas. Given the cross-sectional design, further research is needed to determine whether such strategies improve clinical adherence and outcomes.

## Figures and Tables

**Table 1:** Sociodemographic and professional characteristics of the participants.

Characteristics	Number (n)/Median	Percentage (%)/[IQR]
Age (years), median [IQR]	36	{31–44}
Sex		
Female	204	53.70%
Male	176	46.30%
Professional Title (n = 380)		
Resident	105	27.60%
Specialist	208	54.70%
Faculty member	67	17.60%
Professional Experience		
Residents (n = 105)		
0–2 years	44	41.90%
2–5 years	61	58.10%
Specialists (n = 208)		
0–10 years	129	62.00%
>10 years	79	38.00%
Institution Type (n = 380)		
Training and Research Hospital	143	37.60%
University Hospital	117	30.80%
State Hospital	78	20.50%
Private Hospital	42	11.10%

IQR: Interquartile range, n: Total number of participants.

**Table 2:** Comparison of professional and institutional characteristics according to overall ERAS knowledge levels.

Characteristics	High Knowledge	Moderate Knowledge	Low Knowledge	*p*-Value
	n (%)	n (%)	n (%)	
Overall (n = 380)	107 (28.2)	181 (47.6)	92 (24.2)	-
Professional Title				<0.001
Resident (n = 105)	17 (16.2)	47 (44.8)	41 (39.0)	
Specialist (n = 208)	69 (33.2)	97 (46.6)	42 (20.2)	
Faculty member (n = 67)	21 (31.3)	37 (55.2)	9 (13.4)	
Professional Experience				
Residents (n = 105)				<0.001
0–2 years (n = 44)	1 (2.3)	17 (38.6)	26 (59.1)	
2–5 years (n = 61)	16 (26.2)	30 (49.2)	15 (24.6)	
Specialists (n = 208)				0.044
0–10 years (n = 129)	51 (39.5)	55 (42.6)	23 (17.8)	
>10 years (n = 79)	18 (22.8)	42 (53.2)	19 (24.1)	
Institution Type				0.038
Training and Research Hospital (n = 143)	33 (23.1)	76 (53.1)	34 (23.8)	
University Hospital (n = 117)	29 (24.8)	54 (46.2)	34 (29.1)	
State Hospital (n = 78)	33 (42.3)	28 (35.9)	17 (21.8)	
Private Hospital (n = 42)	12 (28.6)	23 (54.8)	7 (16.7)	

Categorical variables were analyzed using the Chi-Square test. A *p*-value of <0.05 was considered statistically significant.

**Table 3:** Summary of the Lowest- and Highest-Scoring Items from the Knowledge Assessment (n = 380).

Item	Question/Parameter	Correct Answer	Correct Response, n (%)
Lowest correct response rates (<30%)			
17	Cardiac surgery: timing of pregabalin administration	**1 h before**	52 (13.7%)
27	Spinal fusion (1–2 level): goal-directed fluid therapy specifically supported?	**False**	77 (20.3%)
19	Cardiac surgery: target preoperative HbA1c level	**<7%**	78 (20.5%)
4	Oral carbohydrate loading in diabetic patients	**False**	104 (27.4%)
Highest correct response rates (≥96%)			
18	Cardiac surgery: hypoalbuminemia increases perioperative risk	**True**	370 (97.4%)
3	Preoperative fasting time for solid foods	**6 h**	367 (96.6%)
31	Orthopedic surgery: multimodal analgesia routinely recommended	**True**	365 (96.1%)

Correct answers are shown in bold. Items were selected for this summary because their correct response rate was below 30% or among the three highest observed (≥96%). HbA1c: Hemoglobin A1c.

## Data Availability

The data presented in this study are available on request from the corresponding author. The data are not publicly available due to privacy restrictions.

## Supplementary Materials

The following supporting information can be downloaded at: https://www.mdpi.com/article/10.3390/healthcare14172690/s1. Table S1: STROBE Checklist; Table S2: Dependent and Independent Variables and Their Measurement Scales; File S1: ERAS Knowledge Assessment Questionnaire (English Translation).

## Abbreviations

ASA	American Society of Anesthesiologists
BIS	Bispectral Index
CHO	Carbohydrate
CI	Confidence Interval
CME	Continuing Medical Education
CVP	Central Venous Pressure
CVS	Cardiovascular Surgery
ERAS	Enhanced Recovery After Surgery
ESAIC	European Society of Anaesthesiology and Intensive Care
HbA1c	Hemoglobin A1c
I/E	Inspiratory/Expiratory Ratio
IQR	Interquartile Range
IV PCA	Intravenous Patient-Controlled Analgesia
KR-20	Kuder–Richardson 20
NG	Nasogastric
OR	Odds Ratio
PONV	Postoperative Nausea and Vomiting
RSI	Rapid Sequence Intubation
TAP	Transversus Abdominis Plane
TARD	Turkish Society of Anesthesiology and Reanimation
