# Supplementary material for: Knowledge and Awareness of Enhanced Recovery After Surgery (ERAS) Protocols Among Anesthesiologists: A Multicenter Cross-Sectional Survey
Source: Healthcare (Basel). 2026 Aug 24;14(17):2690. doi: 10.3390/healthcare14172690 (PMC13565663; doi:10.3390/healthcare14172690)
Supplement: Supplementary file 1 [file healthcare-14-02690-s001.zip › healthcare-4482985_File S1.pdf]

File S1. ERAS Knowledge Assessment Questionnaire (English Translation)

This document presents the complete 37-item knowledge-assessment questionnaire used in this study, translated into English. The instrument was developed by the research team based on current ERAS Society guidelines and covers general ERAS principles (Items 1–14) and ten surgery-specific modules (Items 15–37). Content and clarity were reviewed by five ERAS-experienced anesthesiology specialists, and the instrument was pilot-tested in 15 anesthesiology and reanimation physicians prior to distribution. The correct answer for each item is indicated in bold and marked "(correct)." This instrument was administered electronically via Google Forms; the original administration language was Turkish.

Part A. General ERAS Principles (Items 1–14)

1. Recommended preoperative fasting time for clear fluids:

- **2 hours (correct)**
- 3 hours
- 4 hours

2. Recommended preoperative fasting time for breast milk:

- 2 hours
- **4 hours (correct)**
- 5 hours
- 6 hours

3. Recommended preoperative fasting time for solid foods:

- 4 hours
- **6 hours (correct)**
- 10 hours

4. Oral carbohydrate (CHO) loading can be applied in diabetic patients.

- True
- **False (correct)**

5. Apfel risk factor: Female sex

- **Yes (correct)**
- No

6. Apfel risk factor: Smoking

- Yes
- **No (correct)**

7. Apfel risk factor: History of postoperative nausea and vomiting (PONV)

- **Yes (correct)**
- No

8. Apfel risk factor: Postoperative opioid use

- **Yes (correct)**
- No

9. Apfel risk factor: Advanced age (>50 years)

- ○ Yes
- ○ **No (correct)**

10. Apfel risk factor: Obesity

- ○ Yes
- ○ **No (correct)**

11. The purpose of oral CHO loading is to decrease insulin resistance.

- ○ **True (correct)**
- ○ False

12. Recommended smoking and alcohol cessation duration before surgery:

- ○ 1–2 weeks
- ○ 2–4 weeks
- ○ **4–8 weeks (correct)**
- ○ 8–12 weeks

13. Recommended timing of oral nutritional supplementation in patients at risk of malnutrition:

- ○ 1–2 days before
- ○ 3–5 days before
- ○ **At least 7 days before (correct)**
- ○ Right before surgery

14. Target intraoperative Bispectral Index (BIS) range:

- ○ 20–40
- ○ **40–60 (correct)**
- ○ 50–70
- ○ 60–80

Part B. Surgery-Specific Modules (Items 15–37)

15. Bariatric surgery: Inverse ratio ventilation (I/E) can be applied intraoperatively.

- ○ **True (correct)**
- ○ False

16. Bariatric surgery: Nasogastric tube and surgical drains should not be used routinely.

- ○ **True (correct)**
- ○ False

17. Cardiac surgery: Recommended timing of pregabalin administration:

- ○ **1 hour before (correct)**
- ○ 4 hours before
- ○ 8 hours before
- ○ 12 hours before

18. Cardiac surgery: Hypoalbuminemia increases perioperative risk.

- ○ **True (correct)**
- ○ False

19. Cardiac surgery: Target preoperative HbA1c level:

- < 6%
- < 6.5%
- **< 7% (correct)**
- < 8%

20. Cardiac surgery: Early extubation is recommended.

- True
- False (correct)

21. Vascular surgery: Preoperative HbA1c measurement is recommended.

- **True (correct)**
- False

22. Colorectal surgery: Routine mechanical bowel preparation is not recommended.

- **True (correct)**
- False

23. Colorectal surgery: Gold-standard analgesia technique:

- IV patient-controlled analgesia (IV PCA)
- **Thoracic epidural analgesia (correct)**
- Lumbar epidural analgesia
- Transversus abdominis plane (TAP) block

24. Colorectal surgery: Intravenous lidocaine infusion reduces opioid consumption.

- **True (correct)**
- False

25. Hepatic surgery: Low central venous pressure (CVP) is targeted during resection.

- **True (correct)**
- False

26. Hepatic surgery: Wound infiltration is equivalent to thoracic epidural analgesia.

- **True (correct)**
- False

27. Spinal surgery: Goal-directed fluid therapy is specifically supported for limited-extent procedures such as 1–2 level lumbar fusion.

- True
- **False (correct)**

28. Neonatal surgery: Routine paracetamol administration reduces opioid requirements.

- **True (correct)**
- False

29. Orthopedic surgery: Routine use of spinal opioids is recommended.

- True
- False (correct)

30. Orthopedic surgery: Tranexamic acid administration reduces perioperative blood loss.

- ○ **True (correct)**
- ○ False

31. Orthopedic surgery: Multimodal analgesia is routinely recommended.

- ○ **True (correct)**
- ○ False

32. Thoracic surgery: Paravertebral block is considered equivalent to thoracic epidural analgesia.

- ○ **True (correct)**
- ○ False

33. Radical cystectomy: Thoracic epidural analgesia is recommended as part of a multimodal analgesia strategy.

- ○ **True (correct)**
- ○ False

34. Emergency laparotomy: Rapid sequence induction/intubation (RSI) is recommended.

- ○ **True (correct)**
- ○ False

35. Emergency laparotomy: Patients are at high risk for postoperative nausea and vomiting (PONV).

- ○ **True (correct)**
- ○ False

36. Emergency laparotomy: Delirium screening is recommended in patients over 65 years.

- ○ **True (correct)**
- ○ False

37. Emergency laparotomy: Avoiding sedative premedication is recommended.

- ○ **True (correct)**
- ○ False
