# Supplementary material for: Knowledge and Awareness of Enhanced Recovery After Surgery (ERAS) Protocols Among Anesthesiologists: A Multicenter Cross-Sectional Survey
Source: Healthcare (Basel). 2026 Aug 24;14(17):2690. doi: 10.3390/healthcare14172690 (PMC13565663; doi:10.3390/healthcare14172690)
Supplement: Supplementary file 1 [file healthcare-14-02690-s001.zip › healthcare-4482985_Tables S1 and S2.pdf]

STROBE Statement

Checklist of items that should be included in reports of cross-sectional studies. Manuscript: "Knowledge and Awareness of Enhanced Recovery After Surgery (ERAS) Protocols among Anesthesiologists: A Multicenter Cross-Sectional Survey''

Table S1. STROBE Checklist.

Item No.	Recommendation	Reported on page/section
1a	Indicate the study's design with a commonly used term in the title or abstract	Title; Abstract ("multicenter cross-sectional survey")
1b	Provide an informative and balanced summary in the abstract	Abstract
2	Explain the scientific background and rationale for the investigation	Introduction
3	State specific objectives	Introduction, final two paragraphs (objectives and a priori hypothesis)
4	Present key elements of study design early in the paper	Methods – Study Design and Ethical Approval
5	Describe the setting, locations, relevant dates (recruitment, exposure, data collection)	Methods – Population and Sample; Data Collection Process (May–Aug 2025)
6a	Give eligibility criteria and sources/methods of participant selection	Methods – Population and Sample
6b	Cross-sectional: N/A (matching not applicable)	N/A
7	Define all outcomes, exposures, predictors, potential confounders, effect modifiers	Methods – Data Collection Tool; Endpoints; Table S2
8	Give sources of data and details of measurement methods for each variable	Methods – Data Collection Tool
9	Describe efforts to address potential sources of bias	Methods – Population and Sample (sampling frame discussion); Limitations
10	Explain how study size was arrived at	Methods – Population and Sample (G*Power calculation)
11	Explain how quantitative variables were handled; describe groupings	Methods – Data Collection Tool (Bloom's cutoff categorization)
12a–e	Describe statistical methods, including subgroup/interaction analyses, missing data handling, sensitivity analyses	Methods – Statistical Analysis (no missing data owing to mandatory online fields, stated explicitly)
13a–c	Report numbers at each stage of the study (eligible, invited, analyzed); give reasons for non-participation; consider a flow diagram	Methods – Population and Sample; Figure 1 (participant flow diagram)
14a	Give characteristics of study participants and information on exposures/confounders	Results; Table 1
14b	Indicate number of participants with missing data	Methods – Statistical Analysis (0 missing; mandatory online fields)
15	Report numbers of outcome events or summary measures	Results; Table 2

Item No.	Recommendation	Reported on page/section
16a–b	Give unadjusted and adjusted estimates with confidence intervals; report category boundaries	Results (ordinal logistic regression ORs with 95% CI; Bloom's cutoff boundaries in Methods)
17	Report other analyses performed (subgroup, sensitivity)	Results (sensitivity linear regression on continuous score)
18	Summarize key results with reference to study objectives	Discussion, opening paragraphs
19	Discuss limitations, including sources of bias and imprecision	Discussion – Limitations
20	Give a cautious overall interpretation considering objectives, limitations, multiplicity, similar studies	Discussion
21	Discuss generalizability of the findings	Discussion (Limitations; Turkey/ESAIC context paragraph)
22	Give source of funding and role of funders	Declarations – Funding

TableS2. Dependent and Independent Variables and Their Measurement Scales

Variable	Role	Measurement Scale	Categories / Range
ERAS knowledge score (continuous)	Dependent (primary outcome)	Interval (ratio-like, 0–100)	0–100, derived from 37 binary-scored items
ERAS knowledge category (Bloom)	Dependent (secondary, descriptive)	Ordinal	Low (<60) / Moderate (60–74) / High (≥75)
Professional title	Independent (covariate)	Nominal	Resident / Specialist / Faculty member
Institution type	Independent (covariate)	Nominal	Training-Research / University / State / Private Hospital
Professional experience	Independent (subgroup variable)	Ordinal	Residents: 0–2 / 2–5 years; Specialists: 0–10 / >10 years
Age	Descriptive characteristic (not modelled)	Continuous (ratio)	Years, reported as median (IQR)
Sex	Descriptive characteristic (not modelled)	Nominal	Female / Male

Note. The continuous knowledge score is the primary outcome measure of the study and was modeled by linear regression; the Bloom-classified category is a descriptive convention and served as the outcome variable of the ordinal logistic regression. The two regression models are reported as complementary rather than hierarchical. Professional title and institution type were entered as covariates in both regression models. Professional experience was examined only within title-stratified subgroups (i.e., separately among residents and among specialists) and was not entered as a covariate in the multivariable models. Age and sex were collected and reported descriptively (Table 1) but were not included as covariates in the regression models.
